# Probiotic DSF counteracts chemotherapy induced neuropathic pain

**DOI:** 10.18632/oncotarget.25524

**Published:** 2018-06-15

**Authors:** Vanessa Castelli, Paola Palumbo, Michele d'Angelo, Nandha Kumar Moorthy, Andrea Antonosante, Mariano Catanesi, Francesca Lombardi, Dalila Iannotta, Benedetta Cinque, Elisabetta Benedetti, Rodolfo Ippoliti, Maria Grazia Cifone, Annamaria Cimini

**Affiliations:** ^1^ Department of Life, Health and Environmental Sciences, University of L’Aquila, L'Aquila, Italy; ^2^ Sbarro Institute for Cancer Research and Molecular Medicine, Department of Biology, Temple University, Philadelphia, USA

**Keywords:** neuropathic pain, inflammation, IL-8

## Abstract

Problem statement: Chemotherapy-induced peripheral neuropathy (CIPN) is a widespread and potentially disabling side effect of various anticancer drugs. In spite of the intensive research focused on obtaining therapies capable to treat or prevent CIPN, the medical demand remains very high. Microtubule-stabilizing agents, among which taxanes, are effective chemotherapeutic agents for the therapy of several oncologic diseases. The inflammatory process activated by chemotherapeutic agents has been interpreted as a potential trigger of the nociceptive process in CIPN. The chemotherapy-driven release of proinflammatory and chemokines has been recognized as one of the principal mechanisms controlling the establishment of CIPN. Several reports have indicated that probiotics are capable to regulate the balance of anti-inflammatory and pro-inflammatory cytokines. Accordingly, it has been suggested that some probiotic formulations, may have an effective role in the management of inflammatory pain symptoms. Experimental approaches used: we tested the hypothesis that paclitaxel-induced neuropathic pain can be counteracted by the probiotic DSF by using an *in vitro* model of sensitive neuron, the F11 cells. On this model, the biomolecular pathways involved in chemotherapy induced peripheral neuropathy depending on inflammatory cytokines were investigated by Real-time PCR, Western blotting and confocal microscopy. General conclusions: the results obtained, i.e. the increase of acetylated tubulin, the increase of the active forms of proteins involved in the establishment of neuropathic pain, point towards the use of this probiotic formulation as a possible adjuvant agent for counteracting CINP symptoms.

## INTRODUCTION

Chemotherapy-induced peripheral neuropathy (CIPN) is a widespread and potentially disabling side effect of various anticancer drugs [1]. The most common symptoms are numbness, tingling or pain, in the feet or hands [1–2]. In a recent meta-analysis of studies involving 4179 patients on various chemotherapeutic treatments, the incidence of CIPN was 68.1% within the first month of treatment, 60.0% at 3 months, and 30.0% at 6 months [3]. CIPN has significant clinical implications that drastically affect life daily, leading to the in the discontinuation of therapy, which could eventually impact overall survival [4, 2]. Despite the intensive research focused on obtaining therapies capable to treat or prevent CIPN [5], the medical demand remains very high. The pathogenesis of CIPN has not been entirely clarified but it is known that the neuropathy symptom profile shows to be strongly shared across various classes of chemotherapeutics comprising platinum compounds, taxanes, vinca-alkaloids, and proteasome inhibitors. Peripheral nerve degeneration or small fiber neuropathy is associated with the main mechanism in the development of CIPN [6–7] but numerous studies underline that the occurrence of neuropathic pain, induced by anticancer drugs, may arise early after the first infusion showing no injury in intra-epidermal nerve fibers or axonal degeneration in peripheral nerves [8–9]. Microtubule-stabilizing agents (MTSAs), among which taxanes, are effective chemotherapeutic agents for the therapy of several oncologic diseases [10]. It is noteworthy that chemotherapeutic agents damage microtubules by interrupting mitochondrial function, or directly targeting DNA can also disturb the integrity and functionality of axons, thus triggering peripheral nerve degeneration. Several preclinical and clinical evidences proposed a shared physio-pathological mechanism partly independent from the antineoplastic drug molecular target. The inflammatory process activated by chemotherapeutic agents has been interpreted as a potential trigger of the nociceptive process in CIPN [11–12] and the chemotherapy-driven release of proinflammatory and chemokines (chemotactic cytokines) has been recognized as one of the principal mechanisms controlling the neuro-immune communication. In fact, chemotherapeutic exposure steadily stimulates production and release of pro-inflammatory cytokines such as TNF-α, IL-1β, IL-6 and chemokines such as IL-8 and MCP-1 [13–15]. Pro-inflammatory cytokines can impact to neural damage not only by triggering the inflammatory process but also by a direct receptor-mediated activity on neurons and glial cells [16–20]. Paclitaxel is a high efficiency, low toxicity, broad spectrum natural plant anticancer medicine. Numerous studies showed that Paclitaxel can inhibit the proliferation of a variety of tumor. Paclitaxel-induced neuropathic pain typically represents sensory neuropathy with the most common symptoms such as numbness, tingling in the hands, feet and burning pain. It is reported that higher doses of Paclitaxel induce axonal degeneration to the peripheral nerves. On the other hand, low dose of Paclitaxel produces hypersensitivity pain including allodynia and hyperalgesia. Neurotoxicity caused by Paclitaxel is attributed to alteration of microtubule structure leading to increased microtubule stability by increasing acetylated α-tubulin causing neuropathic pain, but it has been recently indicated that Paclitaxel triggers the activation of IL-8 signaling in DRG neurons in culture [21].

Several reports have indicated that probiotics are capable to regulate the balance of anti-inflammatory and pro-inflammatory cytokines and to increase immunoglobulins levels, for example IgA. Studies have demonstrated that probiotic formulations are able to modulate the immune system, down-regulating the inflammatory factors of immune system, in particular, inducing decrement of proliferation of the T-Cells, reduction of pro-inflammatory cytokines (including IL-1β, IL-2, IL-6, IL-12, IL-17, IFN-γ, TNF-α), increase of regulatory and anti-inflammatory cytokines (IL-10, TGF-β) and decrease of NF-κB and other intracellular signaling pathways mediators [22–25]. Accordingly, it has been suggested that some probiotic formulations, may have an effective role in the management of inflammatory pain symptoms. [26–27].

A dose-effect relationship with adequate dosage of the probiotic formulation for health benefits. DSF is a high concentration probiotic formulation (450 billion bacteria per sachet), which has been recognized by the main Gastroenterology Associations for the dietary management of pouchitis as well as ulcerative colitis [28].

In this work, we wanted to test the hypothesis that Paclitaxel-induced neuropathic pain can be counteracted by the probiotic formulation of DSF. To this purpose, the biomolecular pathways involved in chemotherapy induced peripheral neuropathy, already described by us in a previous work [21], were investigated.

## RESULTS

Preliminary results were devoted to set the DSF extract concentration showing no toxic effect, evaluated by cell viability dose-dependent curve (Supplementary Figure 1). On the basis of these preliminary data, all the subsequent experiments were performed using 0.1 mg/ml of extract.

**Figure 1:**
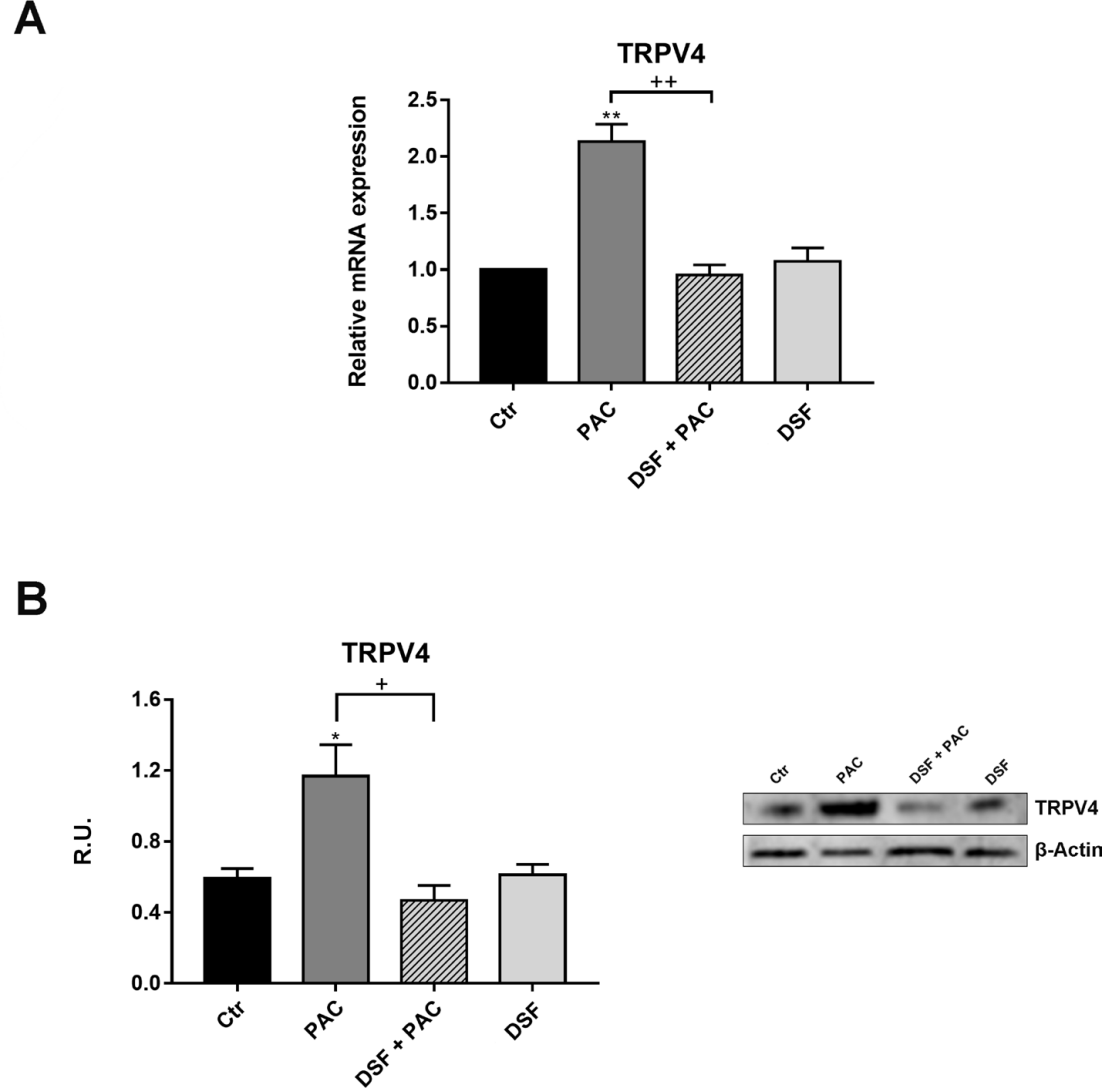
(**A**) Real-time PCR for TRPV4 in control and treated cells. Data are mean ± SE of three different experiments run in triplicate. ^*^p < 0.05; ^**^p < 0.005 versus control values. ^+^*p* < 0.05, ^++^*p* < 0.005 vs pac-treated cells. (**B**) Western blotting and relative densitometric analysis for TRPV4. Data are mean ± SE of three different experiments run in triplicate. ^*^p < 0.05 versus control values. ^+^*p* < 0.05, vs pac-treated cells.

Paclitaxel is known to induce neuropathic pain by increasing the expression of TRPV1 and TRPV4 pain receptors [29–31]. Therefore, the expression of the receptor under Paclitaxel treatment and under the combined treatment Pac-DSF was assayed by Realtime-PCR and Western blotting, the expression of the receptors under Paclitaxel treatment and evaluated the effect of DSF. In Figure 1, the Real-time PCR analysis for TRPV4 on F11 cells under the different treatments is reported (Figure 1A). It possible to observe that Paclitaxel significantly increases TRPV4 mRNA, while the concomitant presence of DSF restores the control conditions. In Figure 1B, the Western blotting analysis for the TRPV4 receptor is reported. TRPV4 protein levels showed the same behavior of the mRNA. In fact, Paclitaxel increases the protein level, while the presence of DSF restores the control conditions.

**Figure 2:**
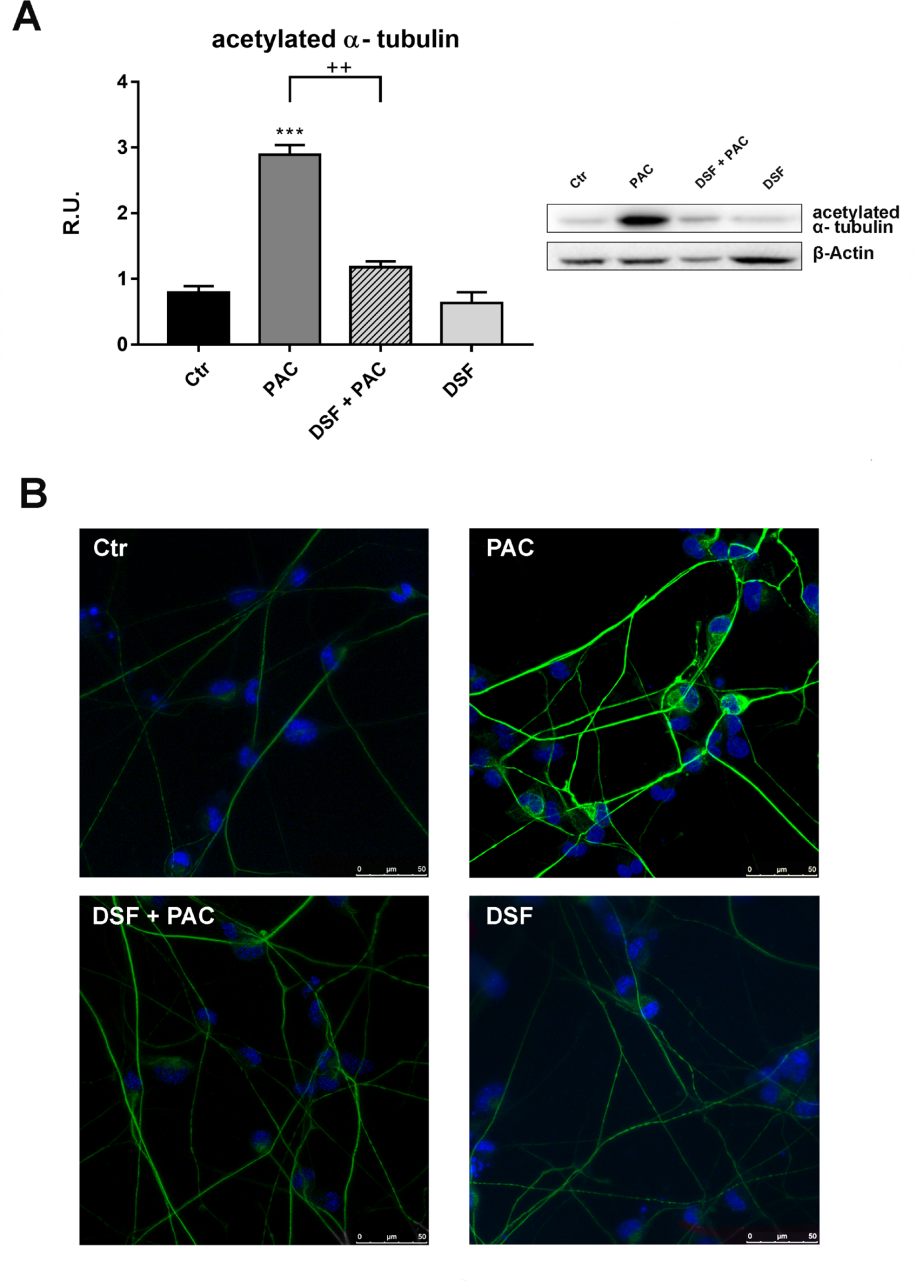
Western blotting for acetylated α-tubulin in control and treated cells In (**A**), Western blotting and relative densitometric analysis for acetylated α-tubulin. A representative blotting is shown. Data are mean ± SE of three different experiments. ^***^p < 0.0005 vs control values. ^++^*p* < 0.005 vs pac-treated cells. In (**B**), Confocal laser microscopy for acetylated α-tubulin in control and treated cells.

It has been previously demonstrated that acetylated α-tubulin increases upon chemotherapy treatment, for this reason the protein was assayed by Western blotting analysis upon the different treatments. In Figure 2A the Western blotting and densitometric analyses for this marker are reported. In agreement with the literature [21–32], Paclitaxel determines a significant increase of acetylated α-tubulin, while under combined treatment (Pac + DSF), the protein appears at the same level of control cells. These results were further confirmed by the immunolocalization experiments for acetylated α-tubulin, where it is possible to observe an increase of the fluorescence intensity upon Paclitaxel treatment and a restore to the control conditions under Pac + DSF (Figure 2B).

**Figure 3:**
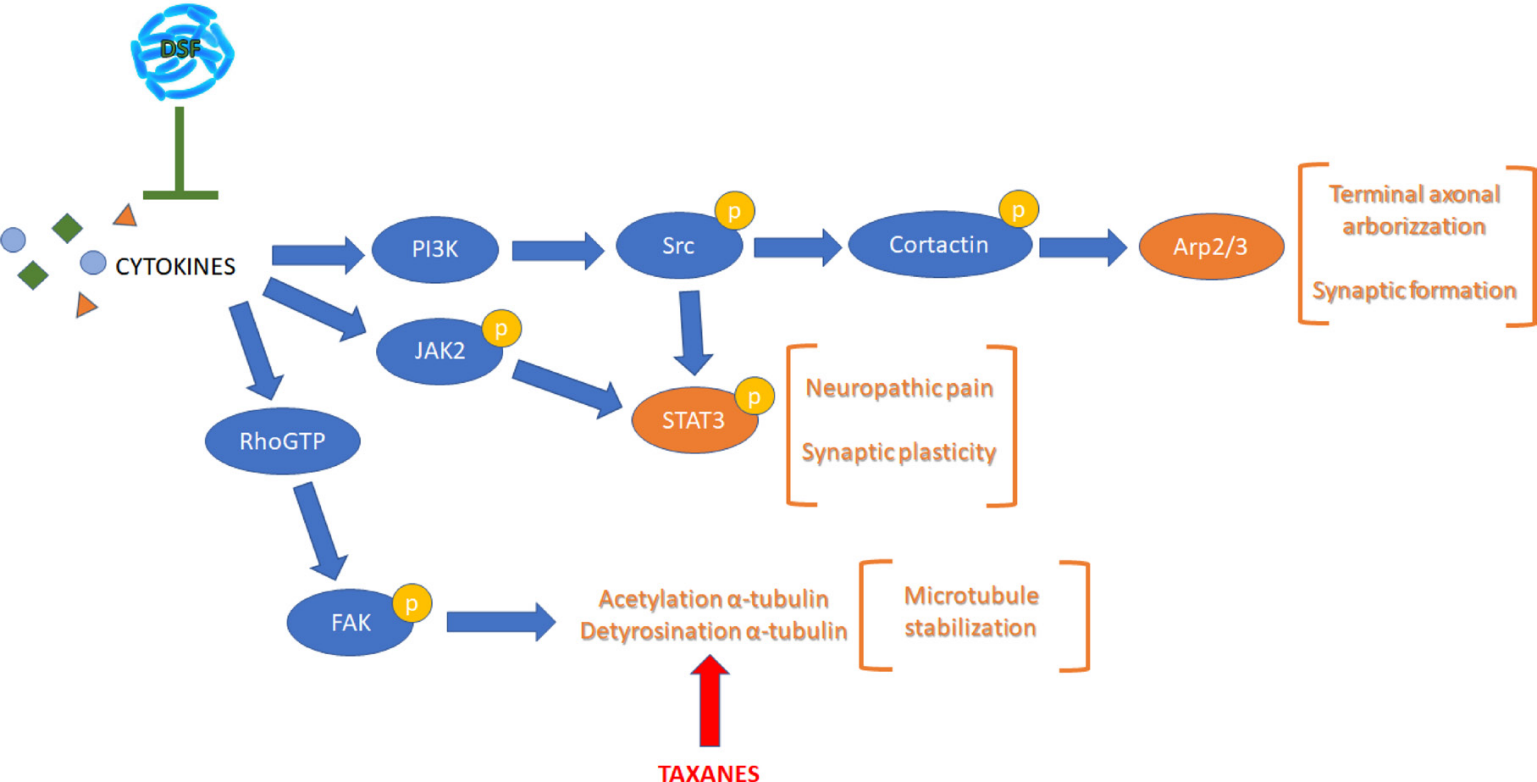
Schematic representation of the pathways considered illustrating most of the proteins analyzed

**Figure 4:**
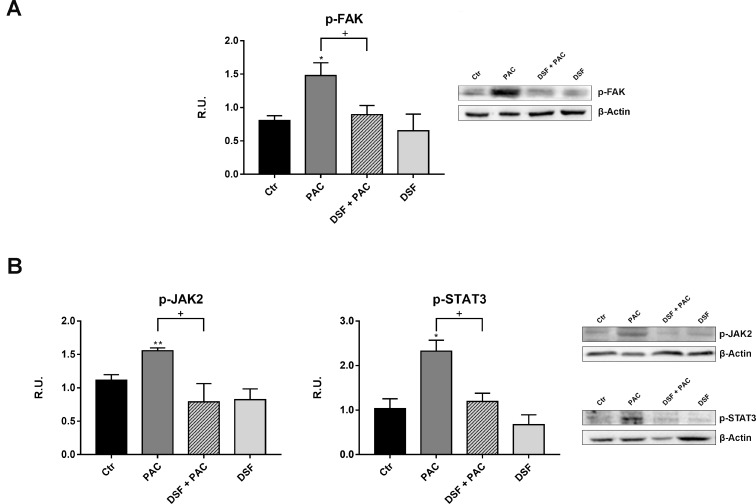
Western blotting and relative densitometric analysis for the signal transduction pathway involved in pain, such as the active forms of FAK (**A**), JAK2, STAT3 (**B**), in control and treated cells. A representative blotting image is shown. Data are mean ± SE of three different experiments. ^*^p < 0.05; ^**^p < 0.005; vs control values. ^+^*p* < 0.05; ^++^*p* < 0.005 vs pac-treated cells.

The subsequent experiments were performed to elucidate the signal transduction pathways involved in the establishment of CINP and possibly mediated by inflammatory cytokines, as depicted in Figure 3, a schematic representation summarizing the pathways controlled by cytokines, such as PI3K, p-JAK2 or p-FAK pathways, all together leading to different aspects of neuropathic pain. To this purpose the first protein analyzed was the active form of the protein of focal adhesion, p-FAK, responsible, once active, of the α-tubulin acetylation. The protein is significantly increased by Paclitaxel treatment, while under combined treatment it shows at the same level of control cells (Figure 4A).

The other enzyme of the analyzed pathway was the active form of the JAK2 protein, involved in the p-STAT3 signaling (Figure 4B), which in turn is involved in neuropathic pain and synaptic plasticity [21, 33–34]. It is possible to observe that Paclitaxel increases p-JAK2, while the presence of DSF extract restores the control conditions. In the same Figure 4B, p-STAT3 levels, analyzed under the different conditions, are reported. Paclitaxel increases p-STAT3 levels with respect to control cells, while DSF counteracts this effect.

**Figure 5:**
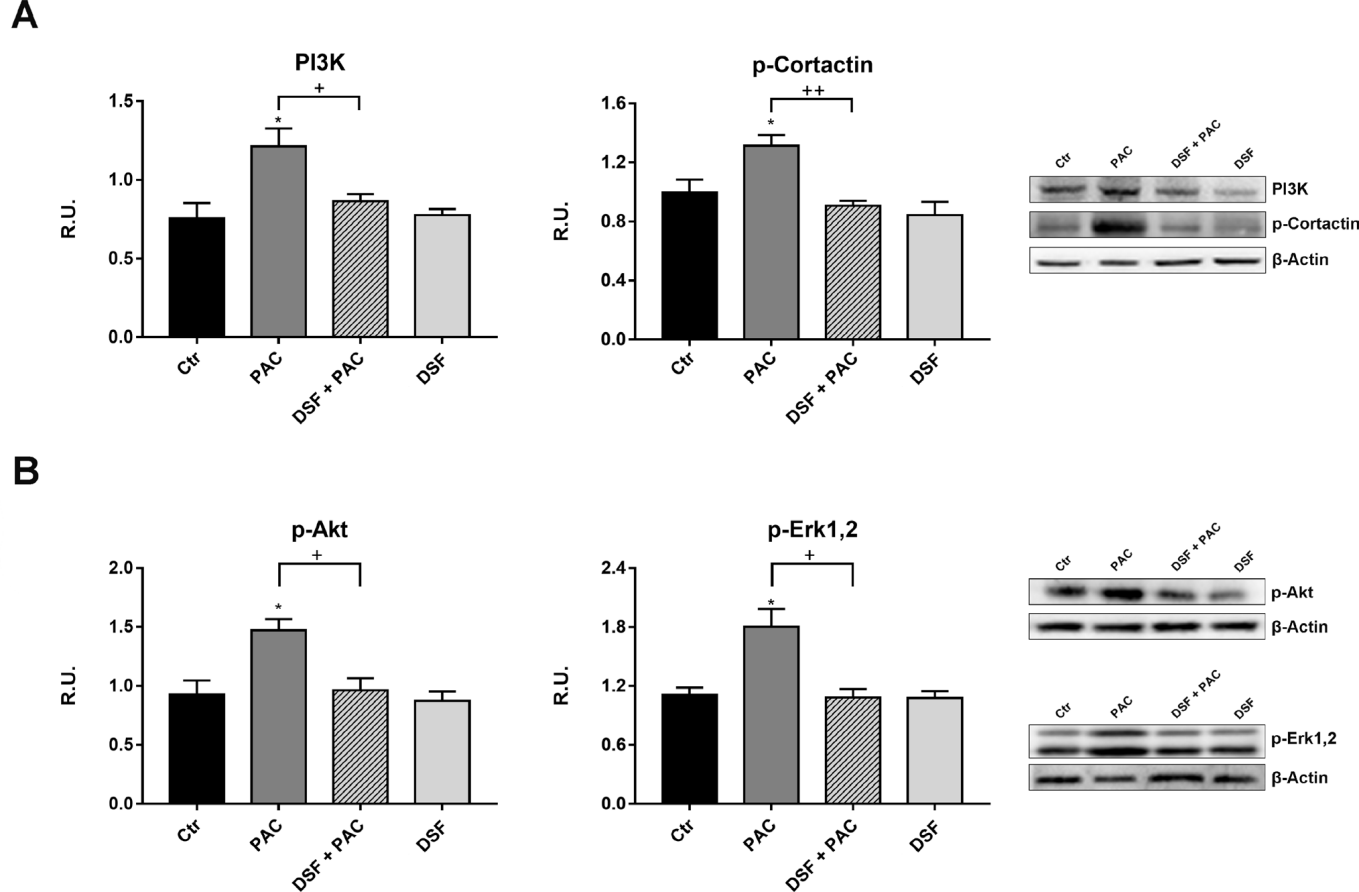
Western blotting and relative densitometric analysis for PI3K and the active form of cortactin in control and treated cells (**A**). In (**B**) Western blotting analysis for p-AKT and p-Erk1,2 in control and treated cells. A representative blotting image is shown. Data are mean ± SE of three different experiments. ^*^p < 0.05; vs control values. ^+^*p* < 0.05; ^++^*p* < 0.005 vs pac-treated cells.

PI3K/p-cortactin pathway, which is crucial for axonal arborization and synaptic plasticity, is strongly up-regulated by paclitaxel, while the presence of the probiotic extract counteracts also this effect (Figure 5A). The PI3K pathways comprises also p-Akt and p-ERK_1,2_; in Figure 5B the behavior of these proteins is reported. In agreement with the activation of PI3K pathway, p-Akt and p-ERK_1,2_ are increased by Paclitaxel, while under the combined treatment the proteins appear at the same level of the controls [35–38].

**Figure 6:**
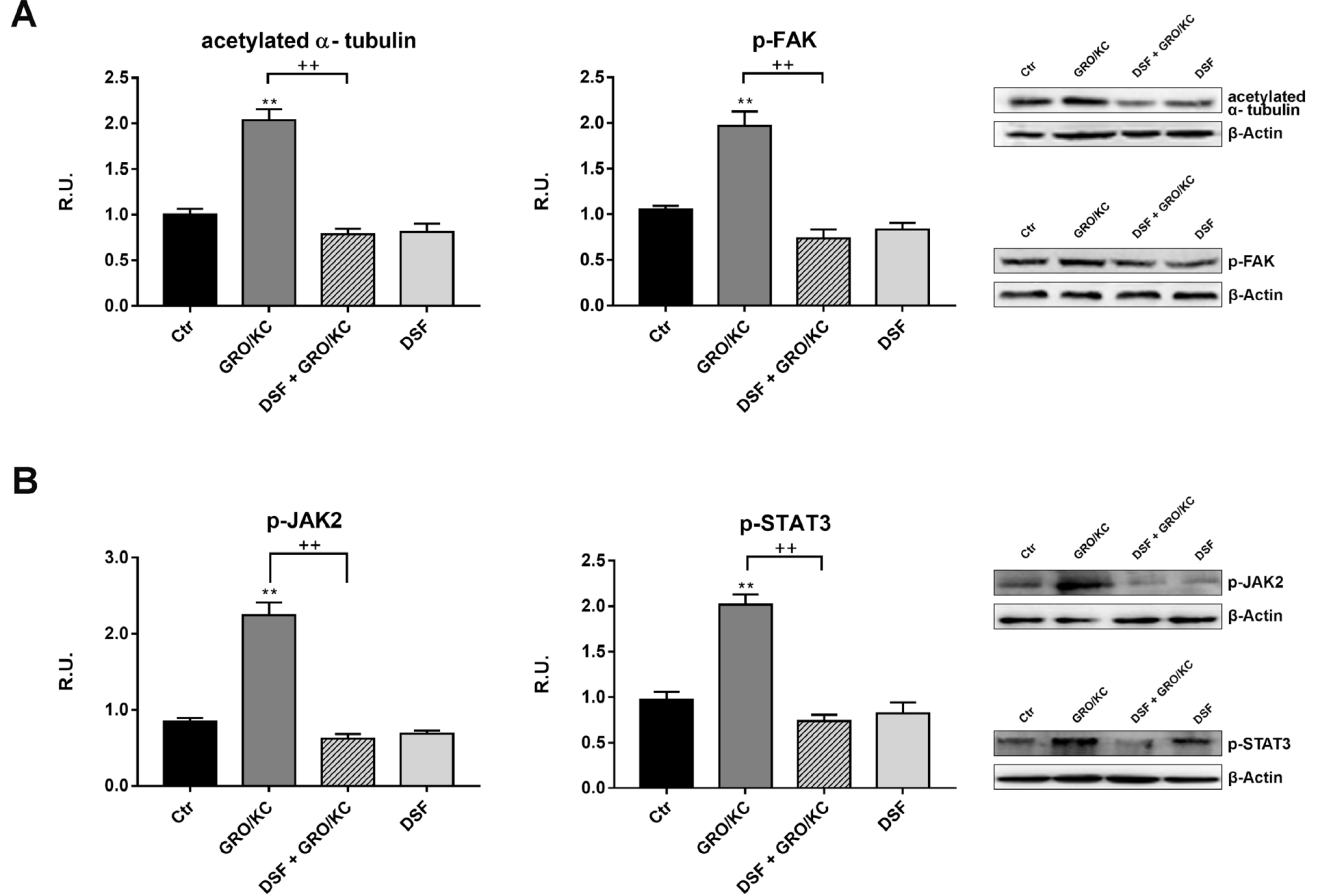
The previous parameters were re-assayed in the presence of GRO/KC in place of Paclitaxel (**A**) Western blotting and relative densitometric analysis for acetylated α-tubulin and p-FAK in control and treated cells. In (**B**), Western blotting and relative densitometric analysis for p-JAK2 and p-STAT3 in control and treated cells. A representative blotting image is shown. Data are mean ± SE of three different experiments. ^**^p < 0.005; ^***^p < 0.0005 vs control values. ^++^*p* < 0.005 vs GRO/KC-treated cells.

**Figure 7:**
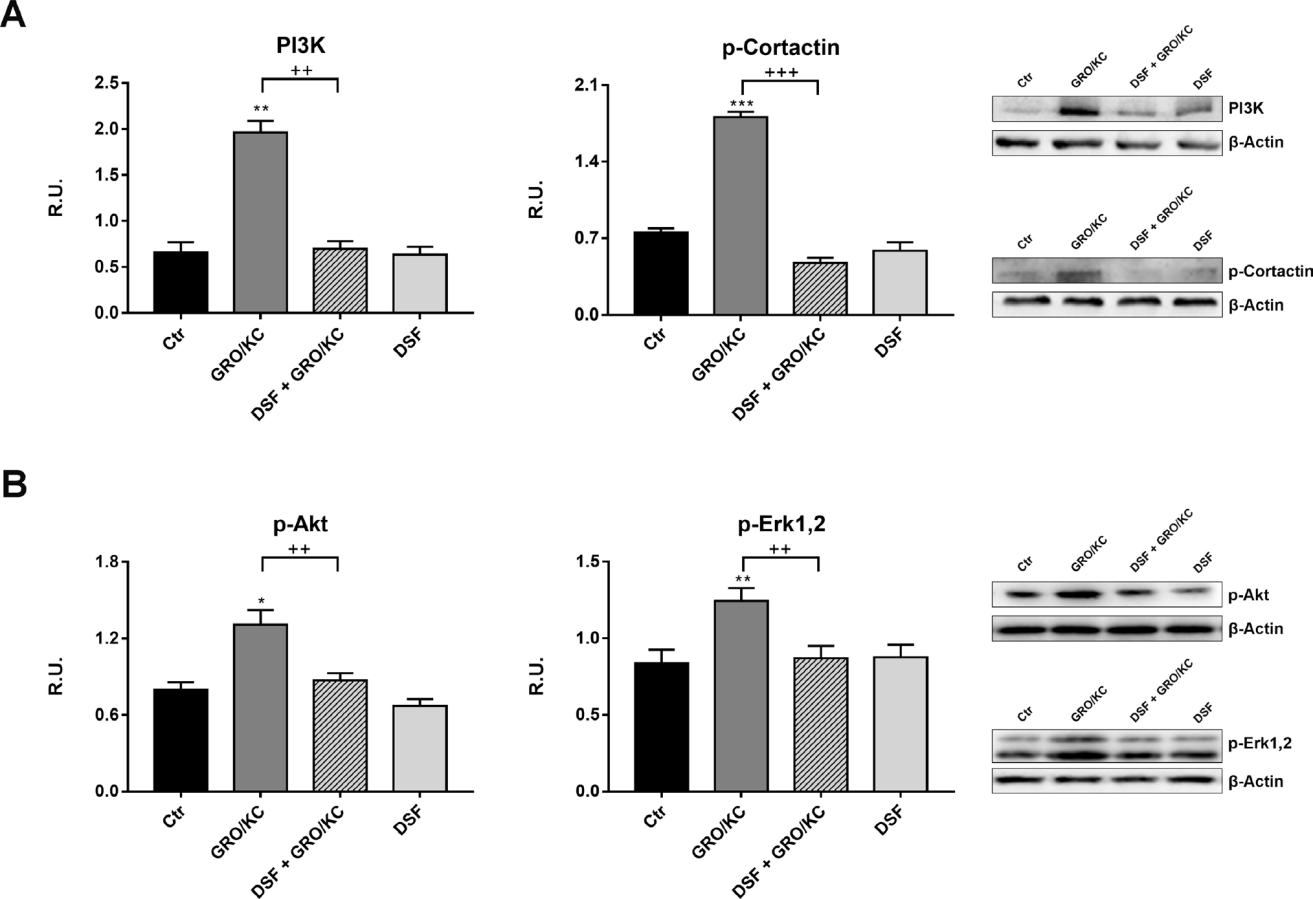
In (**A**), Western blotting analysis for PI3K and P-cortactin in control and treated cells. In (**B**), Western blotting analysis for p-AKT and p-Erk1,2 protein level. A representative blotting image is shown. Data are mean ± SE of three different experiments. ^*^p < 0.05; vs control values. ^+^*p* < 0.05; ^++^*p* < 0.005 vs GRO/KC-treated cells.

Among the chemokines, IL-8 and its receptors CXCR1/2 have been shown to be upregulated in numerous animal models following nerve injury and implicated in development and maintenance of neuropatic pain and of the inflammatory hypernociception. For this reason, in order to demonstrate that DSF treatment may inhibits IL-8-dependent pathways, that it has been previously demonstrated active in our experimental model of neuropathic pain [21], differentiated F11 cells have been treated with GRO/KC (the murine analogous of IL-8) in place of Paclitaxel. In Figure 6 the Western blotting analysis for all parameters previously assayed, under this new experimental condition, is now shown. It is possible to observe that GRO/KC exerts the same effects of Paclitaxel on acetylated α-tubulin and p-FAK (Figure 6A) and that DSF extract is able to counteract these effects, thus indicating that probiotic formulation is active against IL-8 pathway. Furthermore, p-JAK2 and p-STAT3 are increased by GRO/KC and counteracted by DSF (Figure 6B). Finally, also PI3K and p-cortactin behave in the same manner, being increased by GRO/KC and counteracted by probiotic extract (Figure 7A). Consequently, GRO/KC increases p-Akt and p-ERK_1,2_ and DSF preparation restores the control conditions (Figure 7B).

## DISCUSSION

Neuropathy induced by taxane administration has been extensively investigated in preclinical and clinical studies. This event is generally associated with Paclitaxel rather than docetaxel and depends on the dose per treatment cycle, the schedule of treatment, and the duration. No drugs exist so far to prevent or treat CINP, despite research efforts to minimize taxane-induced neurotoxicity which often leads to dose reduction or treatment interruption with a negative impact on patients’ clinical outcome. Several independent reports demonstrated an increase of peripheral pro-inflammatory and chemoattractant cytokines induced by chemotherapy and an evident correlation with peripheral neuropathy [11–39].

Neurons and infiltrated macrophages produce and secrete inflammatory cytokines and chemokines upon chemotherapy that can cause neurotoxicity not only by inducing inflammation but also by modulating spontaneous nociceptor sensitivity and activity [40–41]. Preclinical studies have shown the key role of pro-inflammatory mediators in the development of CINP. For example, the blockage of specific IL-1β and TNFα attenuated peripheral neuropathy in several animal models [16, 38–43]. It has been demonstrated that IL-8 and its receptors CXCR1/2 appears as promising target for the pharmacological management of CINP due to its involvement in sympathetic components of inflammatory hypernociception and in neuropathic nociceptive response [44–45]. In support to this finding, a controlled clinical study indicated a significant up-regulation of IL-8 gene expression in the skin of patients with CIPN [46–47]. Moreover, it has been demonstrated by us that Paclitaxel is able to slightly, but significantly, increase IL-8 release by sensitive neurons in culture [21].

In this paper, we tested for the first time the hypothesis that inhibition of inflammatory signals by probiotics may attenuate, at cellular level, the development of chemotherapy induced neurotoxicity. Starting from the observation, previously reported by us, that IL-8 signal was responsible for paclitaxel-induced neuropathic pain [21] and with the aim to dissect molecular mechanisms underlying the effects of DSF formulation in counteracting initiation and progression of taxane-induced peripheral neuropathy, we also evaluated direct effects of IL-8-induced signaling in DRG derived neurons cultures. The first event in the establishment of neuropathic pain, triggered by inflammation, is the mRNA increase for the pain receptors TRPV1 and 4. We report in this study that Paclitaxel upregulate the receptors and DSF extract is able to counteract this effect.

Tubulin acetylation contributes in the multifaceted process regulating microtubule dynamics that is influenced by several microtubule-associated proteins (MAPs). In our experimental conditions, the Paclitaxel-induced increase of α-acetylated tubulin, in DRG derived neurons, was efficiently counteracted by the presence of DSF extract. We also confirmed our previous findings reporting the Paclitaxel effects in increasing parameters involved in neuropathic pain, such as p-STAT3, p-Cortactin, PI3K, p-FAK, p-JAK2. The addition of DSF extract in paclitaxel-treated DRG neurons, restored the control conditions, thus indicating that this probiotic formulation is able to counteract the inflammatory signals in CINP. In fact, the data obtained utilizing GRO/KC (the murine analogous of IL-8) strongly indicated that DSF extraction effectively inhibited *in vitro* paclitaxel-induced neurotoxic effects. On the basis of the results obtained, it is possible to suggest that DSF formulation is able to counteract Paclitaxel-induced neuropathic pain related to IL-8 signaling and that the use of this specific probiotic formulation may represent a valid adjuvant agent to Paclitaxel, useful and not toxic for long lasting therapies.

## MATERIALS AND METHODS

### Cell culture

The F11 hybridoma cells (ECACC 08062601) was chosen as model of dorsal root ganglion (DRG) neurons. They were cultured in DMEM (Euroclone, MI, Italy) medium supplemented with 10% FBS (Sigma-Aldrich St. Louis, CO, USA), 1% penicillin/streptomycin (Euroclone) and 1% glutamine (Euroclone) at 37° C, in a humidified 95% air-5% CO2 atmosphere. For all the experiments cells were used at 18th passage.

Cells were differentiated with rat NGF (rNGF) (from Sigma-Aldrich St. Louis, CO, USA). rNGF was prepared dissolving in DMEM with 1% penicillin/streptomycin and 1% glutamine (FBS free) at the final concentration of 50 ng/ml. Medium was replaced every 3 days until complete differentiation, that happened after 7 DIV.

Following neuronal differentiation, neurons were treated for 24 hours with paclitaxel (Sigma- Aldrich; 10 nM final concentration) or GRO/KC (Peprotech, stock solution 0,1 mg/ml; final concentration 20 ng/ml) DSF (lot.DM538) (0.1 mg/ml) and the combination of this latter + Paclitaxel or GRO/KC. After treatment, the Petri plates containing cells were dehydrated and stored at −20° C for overnight. The Petri plates were taken from −20° C and the cells were scrapped from the plates and treated with ice cold RIPA buffer to extract the protein from the cells. After protein extraction, the eppendorf tubes containing the protein were centrifuged at 4° C and the protein concentration was measured using the BCA protein assay kit.

### Preparation of bacterial extracts

The bacterial extracts were prepared from DSF (DeSimoneformulation: Vivomixx in EU; Visbiome in USA, kindly donated by Prof Claudio De Simone) contains the following bacterial strains: L. plantarum DMS24730, S. thermophilus DSM24731, B. breve DSM24732, L. paracasei DSM24733, L. delbrueckii subsp. Bulgaricus DSM24734, L. acidophilus DSM24735, B. longum DSM24736, B. infantis DSM24737.

For bacterial extract preparation, stocks of 1 g of DSF formulation (lot. DM531) suspended in 10 ml of Phosphate Buffer Saline (PBS, Euroclone, West York, UK) were centrifuged at 8,600 × g, washed twice, resuspended in 10 ml of PBS and sonicated (30 min, alternating 10 secs of sonication and 10 secs of pause) using Vibracell sonicator (Sonic and Materials, Danbury, CT). Bacterial cell disruption was verified measuring the absorbance of the samples at 590 nm (Eppendorf Hamburg, Germany) before and after every sonication step. The sonication steps were repeated until the optical density reached a constant value. Samples were then filtered using 0.22 μm pore filter (Corning Incorporated, Corning, NY, USA) to remove whole bacteria remaining.

### Protein assay

Protein concentrations were assayed by the Pierce BCA Protein Assay kit (Thermo scientific, Waltham, USA). Briefly, this assay is a detergent-compatible formulation based on bicinchoninic acid (BCA) for the colorimetric detection and quantitation of total protein. The method combines the reduction of Cu^2^ to Cu^1^ by protein in alkaline medium (the biuret reaction) with the high sensitive and selective colorimetric detection of the cuprous cation by using a reagent containing BCA. The purple-colored reaction product of this assay is formed by the chelation of two molecules of BCA with one cuprous ion. This complex exhibits a strong absorbance at 562 nm.

### MTS assay

Cell viability was determined using Cell Titer One Solution Cell Proliferation Assay (Promega Corporation Madison, WI, USA) a colorimetric method based on 3-(4,5-dimethylthiazol-2-yl)-5-(3-carboxymethoxyphenil)-2-(4-sulfophenyl)-2H-tetrazolium (MTS). The quantity of formazan formed, as a function of viability, was measured at 492 nm using an ELISA plate reader, Infinite F200 (Tecan, Männedorf, Swiss). The assay was performed in quintuplicate. The results were expressed as absorbance at 492 nm.

### Immunofluorescence

Cells were fixed in 4% paraformaldehyde in PBS for 20 min at room temperature (RT) and permeabilized in methanol for 5 min at −20° C. Cells were then blocked with PBS containing 4% BSA for 30 min and incubated with the following primary antibodies diluted in the blocking solution overnight at 4° C: rabbit acetylated α-tubulin (Cell Signaling, Danvers, USA) 1:1000. Cells were then rinsed in PBS several times before incubation with secondary antibodies, goat anti-rabbit conjugated with Alexafluor 633 (1:2000), for 30 min at RT. After extensive washing, coverslips were mounted with Vectashield mounting medium with DAPI (Vector Laboratories Burlingame, CA, USA) and then observed at confocal laser microscope (Leica, Wetzlar, Germany).

### Western Blotting

Control and treated cells were collected and lysated in ice-cold RIPA buffer (phosphate buffer saline pH 7.4 containing 0.5% sodium deoxycolate, 1% Igepal, 0.1% SDS, 5mM EDTA, 1% protease and phosphatase inhibitor cocktails, Sigma). Protein lysates (30 μg) were separated on 9–10% SDS–polyacrilamide gel and electroblotted on to polyvinyldifluoride membrane (PVDF; Sigma, St Loius, USA). Nonspecific binding sites were blocked by 5% non-fat dry milk (Bio-Rad Laboratories, Hercules, CA) in Tris buffered saline (TBS: 20 mMTris– HCl, pH 7,4, containing 150 mM NaCl) for 45 minutes at RT. Afterwards, membranes were incubated overnight at 4° C with the following primary antibodies, diluted with TBS containing 0,1% Tween 20 (TBS-T) and 5% non-fat dry milk: mouse acetylated α-tubulin 1:1000 (Cell Signaling), rabbit p-FAK 1:1000 (Cell Signaling), rabbit p-JAK2 1:200 (Santa Cruz, Dallas, USA), rabbit PI3K 1:500 (Cell Signaling), rabbit p-Cortactin 1:1000 (Abcam, Cambridge, UK), goat pSTAT3 1:100 (Santa Cruz, Dallas, USA) and HRP-conjugated Actin 1:10000 (Cell Signaling). As secondary antibodies, peroxidase conjugated anti-rabbit or anti-mouse IgG 1:10000 (KPL, Sera care) and anti-goat 1:4000 (Santa Cruz) were used. Immunoreactive bands were visualized by ECL (Thermo scientific, Waltham, USA), according to the manufacturer's instructions. The relative densities of the immunoreactive bands were determined and normalized with respect to Actin, using ImageJ software. Values were given as relative units (RU).

### Real-time PCR

Total RNA was extracted by Trizol reagent (Life Technologies Lofer, Salzburg, Austria), according to the manufacturer's instructions. The total RNA concentration was determined spectrophotometrically in RNAase-free water, and 1 μg aliquots of total RNA were reverse transcribed into cDNA using ProtoScript First Strand cDNA Syntesis Kit (New England BioLabs, Ipswich, MA). RT-PCR was carried out on ABI 7300HT sequence detection system (ABI), in a total volume of 20 μl containing EagleTaq Universal Master Mix (ROX) (Roche, Penzberg, Germany), DEPC water, 5 μl of cDNA. The following Prime Time qPCR Assay: TRPV1 qRnoCIP0024978 and TRPV4 qRnoCIP0027857 were purchased from Biorad (Biorad Laboratories; Hercules, California, USA). Quadruplicate samples were run for each gene. The reference gene GAPDH qRnoCIP0050838 were used as an internal control to normalize the expression of target genes. Relative expression levels were calculated for each sample after normalization against reference gene, using the ΔΔCt method for comparing relative fold expression differences, as previously described [48].

### Statistics

For the results, data were expressed as mean ± SE. Statistical analysis was performed by the ttest analysis (two tails). The level of significance was set at *P* < 0.05.

## SUPPLEMENTARY MATERIALS AND FIGURES


