# Supplementary material for: Probiotic DSF counteracts chemotherapy induced neuropathic pain
Source: Oncotarget. 2018 Jun 15;9(46):27998–8008. doi: 10.18632/oncotarget.25524 (PMC6021327; doi:10.18632/oncotarget.25524)
Supplement: Supplementary file 1 [file oncotarget-09-27998-s001.pdf]

Probiotic DSF counteracts chemotherapy induced neuropathic pain

SUPPLEMENTARY MATERIALS

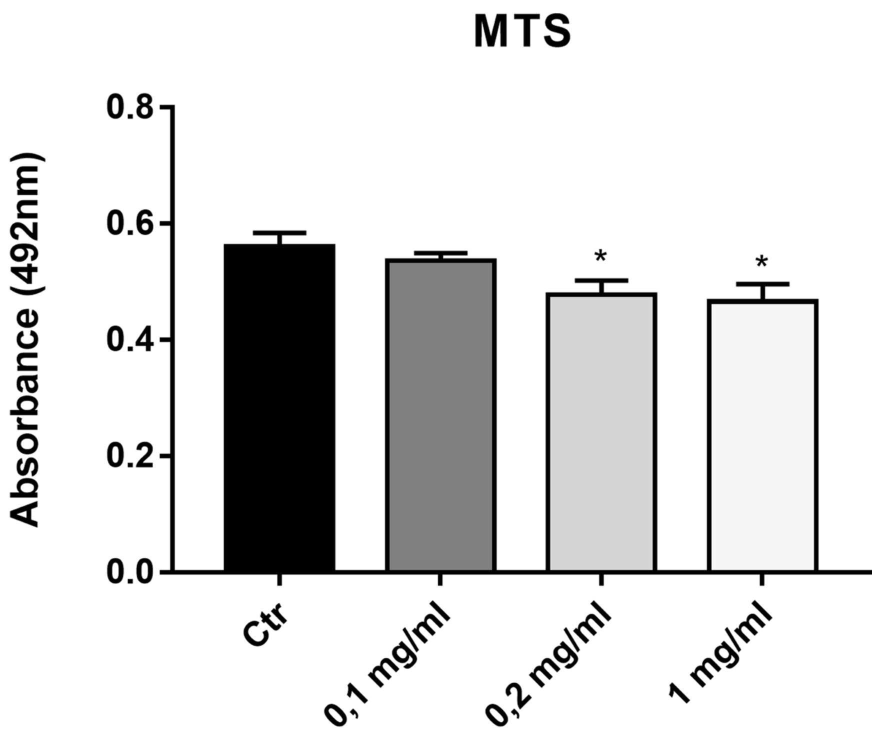

Supplementary Figure 1: MTS assay in differentiated F11 cells treated with different concentrations of DSF extract. Data are mean ± SE of three different experiments. *, $p < 0.05$.
